# Usefulness of myeloperoxidase as a biomarker for the ranking of pulmonary toxicity of nanomaterials

**DOI:** 10.1186/s12989-018-0277-x

**Published:** 2018-10-23

**Authors:** Taisuke Tomonaga, Hiroto Izumi, Yukiko Yoshiura, Toshihiko Myojo, Takako Oyabu, Byeong-Woo Lee, Takami Okada, Takashi Marui, Ke-Yong Wang, Masaru Kubo, Manabu Shimada, Shingo Noguchi, Chinatsu Nishida, Kazuhiro Yatera, Yasuo Morimoto

**Affiliations:** 1 Institute of Industrial Ecological Sciences, University of Occupational and Environmental Health, Kitakyushu, 807-8555 Japan; 2 Shared-Use Research Center, School of Medicine, University of Occupational and Environmental Health, Kitakyushu, 807-8555 Japan; 3 Department of Chemical Engineering, Hiroshima University, Higashi-Hiroshima, 739-8528 Japan; 4 Department of Respiratory Medicine, University of Occupational and Environmental Health, Kitakyushu, 807-8555 Japan

**Keywords:** Myeloperoxidase, Nanomaterial, Pulmonary toxicity, Biomarker, Intratracheal instillation, Inhalation, Rat

## Abstract

**Background:**

In order to examine whether myeloperoxidase (MPO) can be a useful marker for evaluating the pulmonary toxicity of nanomaterials, we analyzed MPO protein in bronchoalveolar lavage fluid (BALF) samples obtained from previous examinations of a rat model. In those examinations we performed intratracheal instillation exposures (dose: 0.2–1.0 mg) and inhalation exposures (exposure concentration: 0.32–10.4 mg/m^3^) using 9 and 4 nanomaterials with different toxicities, respectively. Based on those previous studies, we set Nickel oxide nanoparticles (NiO), cerium dioxide nanoparticles (CeO_2_), multi wall carbon nanotubes with short or long length (MWCNT (S) and MWCNT (L)), and single wall carbon nanotube (SWCNT) as chemicals with high toxicity; and titanium dioxide nanoparticles (TiO_2_ (P90) and TiO_2_ (Rutile)), zinc oxide nanoparticles (ZnO), and toner with external additives including nanoparticles as chemicals with low toxicity. We measured the concentration of MPO in BALF samples from rats from 3 days to 6 months following a single intratracheal instillation, and from 3 days to 3 months after the end of inhalation exposure.

**Results:**

Intratracheal instillation of high toxicity NiO, CeO_2,_ MWCNT (S), MWCNT (L), and SWCNT persistently increased the concentration of MPO, and inhalation of NiO and CeO_2_ increased the MPO in BALF. By contrast, intratracheal instillation of low toxicity TiO_2_ (P90), TiO_2_ (Rutile), ZnO, and toner increased the concentration of MPO in BALF only transiently, and inhalation of TiO_2_ (Rutile) and ZnO induced almost no increase of the MPO. The concentration of MPO correlated with the number of total cells and neutrophils, the concentration of chemokines for neutrophils (cytokine-induced neutrophil chemoattractant (CINC)-1 and heme oxygenase (HO)-1), and the activity of released lactate dehydrogenase (LDH) in BALF. The results from the receiver operating characteristics (ROC) for the toxicity of chemicals by the concentration of MPO proteins in the intratracheal instillation and inhalation exposures showed that the largest areas under the curves (AUC) s in both examinations occurred at 1 month after exposure.

**Conclusion:**

These data suggest that MPO can be a useful biomarker for the ranking of the pulmonary toxicity of nanomaterials, especially at 1 month after exposure, in both intratracheal instillation and inhalation exposure.

**Electronic supplementary material:**

The online version of this article (10.1186/s12989-018-0277-x) contains supplementary material, which is available to authorized users.

## Background

Nanomaterials are defined as having a size of less than 100 nm in one of their three dimensions [1]. New nanomaterials are being produced produced daily and are being used in various fields through the development of nanotechnology. These nanomaterials are useful in cutting-edge technology, but some reports claim that they are toxic even in small amounts and can easily migrate to multiple organs. Some of the materials are fibrous like asbestos, and those with high pulmonary retention are considered to cause induced asbestos-related lung disease, raising concern that nanomaterials may have negative effects on the human body [2–6].

It is important to evaluate the pulmonary toxicity of nanomaterials. In pulmonary toxicity caused by inhaled chemicals including nanomaterials, it is generally thought that the chemicals penetrate into the lung and cause repeated inflammation; in other words, persistent inflammation causes irreversible lesions such as a fibrosis and tumor [7–10]. It is known that silica and asbestos, with high levels of toxicity, cause persistent inflammation, fibrosis and tumor [11, 12]. In contrast, it is reported that titanium dioxide and fullerene, with low levels of toxicity, result in only transient inflammation and no irreversible lesions. Therefore it is thought that pulmonary inflammation, such as a persistent inflammation, is an important process in the induction of irreversible lesions such as fibrosis and tumor [7–10].

Myeloperoxidase (MPO) is one of the degrading enzymes mostly produced by neutrophils, and it is regarded as a main attack factor against foreign bodies by neutrophils [13]. Considering that most inflammation induced by inhaled chemicals is neutrophil inflammation, it is thought that MPO is directly and deeply involved in lung injury and inflammation, and some reports assert that MPO might be a useful biomarker for evaluating the pulmonary toxicity of inhaled chemicals. Haegens et al [14] reported that asbestos-associated lung inflammation in myeloperoxidase-null mice was lower than that in normal asbestos-exposed mice. In terms of exposure to inhaled chemicals such as toner, Fe_2_O_3_, or Ag supported SiO_2_, there are some reports that the concentration of MPO protein in bronchoalveolar lavage fluid (BALF) increases along with lung inflammation [15, 16].

In order to investigate whether MPO related to inflammation caused by oxidative stress can be an useful marker for evaluating pulmonary toxicity of nanomaterials in respiratory exposure examinations, we analyzed MPO in BALF samples obtained from intratracheal instillation and inhalation exposures using nanomaterials with differing toxicities.

## Results

### Characterization of chemicals including nanomatrials

In the present study we used nanoparticles of nickel oxide (NiO), two types of titanium dioxide (TiO_2_ (P90) and TiO_2_ (Rutile)), cerium dioxide (CeO_2_), zinc oxide (ZnO), single wall carbon nanotubes (SWCNT), multi wall carbon nanotubes of different lengths (MWCNTs: MWCNT (S) and MWCNT (L)), and toner with external additives including nanoparticles. The physicochemical profiles of these samples are shown in Table 1. The data of these samples have been published in previous studies [17–25]. We defined the toxicity of the chemicals as follows: in intratracheal instillation studies, the chemicals which induced either persistent inflammation, fibrosis or tumor were set as having high toxicity, and the chemicals that did not induce any of those pathological conditions were set as having low toxicity. Accordingly, NiO, CeO_2_, SWCNT, MWCNT (S), and MWCNT (L) were classified as chemicals with high toxicity, and TiO_2_ (P90), TiO_2_ (Rutile), ZnO, and toner were classified as chemicals with low toxicity.

**Table 1:** Characterization of inhaled chemicals including nanomaterials

Samples	Toxicity	Exposure route	Characterization	Animal (rats)	Negative control	Dose / Concentration	Reference
NiO	High	IT / IH	Size 19 nm, BET 57 m^2^/g Secondary particle diameter (DLS)59.7 nm	Male Fischer344	Distilled water/Clean air	0.2 mg, 1.0 mg/0.32 ± 0.07 mg/m^3^, 1.65 ± 0.20 mg/m^3^	Morimoto et al [18] Oyabu et al [19]
CeO_2_	High	IT / IH	Size 7.8 nm, BET 101 m^2^/g Secondary particle diameter (DLS)10.0 nm	Male Fischer344	Distilled water/Clean air	0.2 mg, 1.0 mg/2.09 ± 0.29 mg/m^3^, 10.2 ± 1.38 mg/m^3^	Morimoto et al [20]
SWCNT	High	IT	Diameter 1.8 nm BET 878 m^2^/g	Male Wistar	Distilled water +0.1% Triton X-100	0.2 mg, 0.4 mg	Morimoto et al [24]
MWCNT (S)	High	IT	Diameter 48 nm Length 0.94 μm	Male Wistar	Distilled water +0.05% Triton X-100	0.2 mg, 1.0 mg	Lee et al [22]
MWCNT (L)	High	IT	Diameter 48 nm Length 3.4 μm	Male Wistar	Distilled water +0.05% Triton X-100	0.2 mg, 0.6 mg	Lee et al [22]
TiO_2_(P90)	Low	IT	Size 14 nm, BET 104 m^2^/g Secondary particle diameter (DLS)22.7 nm	Female Wistar	Distilled water	0.2 mg, 1.0 mg	Yoshiura et al [17]
TiO_2_(Rutile)	Low	IT / IH	Size 12 nm × 55 nm, BET 111 m^2^/g Secondary particle diameter (DLS)44.9 nm	Male Fischer344	Distilled water/Clean air	0.2 mg, 1.0 mg/0.50 ± 0.26 mg/m^3^, 1.84 ± 0.74 mg/m^3^	Morimoto et al [18] Oyabu et al [19]
ZnO	Low	IT / IH	Size 35 nm, BET 31 m^2^/g Secondary particle diameter (DLS)33 nm	Male Fischer344	Distilled water/Clean air	0.2 mg, 1.0 mg/2.11 ± 0.45 mg/m^3^, 10.4 ± 1.39 mg/m^3^	Morimoto et al [21]
Toner	Low	IT	Size 4.05 μm, BET 2–3 m^2^/g Including nanoparticles as external additives(Titanium dioxide and amorphous silica)	Female Wistar	Distilled water +0.1% Tween-80	1.0 mg	Morimoto et al [25]

*IT* intratracheal instillation, *IH* inhalation exposure

### Cell analysis in BALF and pathological features in the rat lung

Table 2 shows summaries of the neutrophil counts in BALF and the pathological features in the rat lung. There were persistent increase in the neutrophil counts in the BALF and persistent inflammation in pathological samples in the chemicals with high toxicity, such as NiO, CeO_2_, MWCNT (S), MWCNT (L), and SWCNT. These results were consistent with our previous studies [17–25].

**Table 2:** Summaries of the neutrophil counts in BALF and pathological features in the rat lung

(A) Intratracheal instillation		3 days	1 week	1 month	3 months	6 months
Pathological featurefeature						
Control (distilled water)		–	–	–	–	–
NiO	0.2 mg	–	+	-~±	–	+
NiO	1.0 mg	+	+	+	–	–
Control (distilled water)		-~±	-~±	-~+	±~+	–
TiO_2_ (P90)	0.2 mg	-or+	-~+	–	+~++	±
TiO_2_ (P90)	1.0 mg	+	-~+	-~+	±	±
Control (distilled water)		–	–	–	–	-~±
TiO_2_ (Rutile)	0.2 mg	–	–	–	–	–
TiO_2_ (Rutile)	1.0 mg	–	–	–	–	–
Control (distilled water)		–	–	–	–	–
CeO_2_	0.2 mg	–	–	–	–	–
CeO_2_	1.0 mg	–	–	–	–	–
Control (distilled water)		–	–	–	–	–
ZnO	0.2 mg	+	±	–	–	–
ZnO	1.0 mg	++	±	-~±	–	–
Control (Triton 0.1%)		-~±	-~±	–	±~+	–
SWCNT	0.2 mg	±	±	-~+	±~++	-~+
SWCNT	0.4 mg	±	±~+	±~+	+	±~+
Control (Triton 0.05%)		±	-~±	-~±	-~±	-~±
MWCNT (Short)	0.2 mg	+	+	±	±	-~±
MWCNT (Short)	1.0 mg	+~++	±~+	±	±	±
Control (Triton 0.05%)		–	-~±	–	±~++	-~+
MWCNT (Long)	0.2 mg	+~++	±	-~±	±~++	±~+
MWCNT (Long)	0.6 mg	+~++	±	–	-~+	±~+
Control (Tween80 0.1%)		–	–	–	–	–
Toner	1.0 mg	±	–	–	–	–
Neutrophil counts in BALF (1000 cells/mL ± SD)						
Control (distilled water)		2.88 ± 1.58	0.22 ± 0.49	0.20 ± 0.45	0.08 ± 0.17	0.69 ± 1.53
NiO	0.2 mg	21.1 ± 8.74**	78.93 ± 18.40**	52.38 ± 12.34**	13.03 ± 12.13**	1.27 ± 1.97
NiO	1.0 mg	153.5 ± 44.6**	158.51 ± 56.43**	161.69 ± 67.27**	279.80 ± 125.57**	59.80 ± 15.28**
Control (distilled water)		0.22 ± 0.25	0.20 ± 0.22	0.26 ± 0.47	0.25 ± 0.35	1.13 ± 1.19
TiO_2_ (P90)	0.2 mg	0.80 ± 0.55	0.31 ± 0.21	0.65 ± 0.46	0.53 ± 0.50	2.36 ± 3.82
TiO_2_ (P90)	1.0 mg	54.50 ± 31.86	20.35 ± 13.61**	2.89 ± 1.97*	0.32 ± 0.35	0.49 ± 0.42
Control (distilled water)		1.73 ± 10.6	0.80 ± 0.73	4.74 ± 2.08	0.57 ± 0.80	0.28 ± 0.39
TiO_2_ (Rutile)	0.2 mg	11.95 ± 4.94**	1.61 ± 1.29	1.81 ± 1.13*	11.07 ± 22.57	1.62 ± 2.88
TiO_2_ (Rutile)	1.0 mg	174.7 ± 121.8**	110.33 ± 39.14**	5.27 ± 0.98	105.21 ± 229.82	0.35 ± 0.51
Control (distilled water)		6.50 ± 5.17	1.76 ± 0.96	0.90 ± 1.32	0.45 ± 0.49	1.37 ± 2.26
CeO_2_	0.2 mg	111.66 ± 48.09**	119.52 ± 79.79**	27.17 ± 15.26**	10.93 ± 3.25**	1.91 ± 1.39
CeO_2_	1.0 mg	170.52 ± 35.04**	234.21 ± 55.62**	74.65 ± 6.72**	56.73 ± 15.54**	11.07 ± 1.88**
Control (distilled water)		2.35 ± 0.41	0.47 ± 0.36	1.08 ± 0.32	1.12 ± 0.81	3.55 ± 1.92
ZnO	0.2 mg	191.38 ± 42.19**	7.032 ± 1.61**	1.02 ± 0.94	3.21 ± 2.23	3.10 ± 1.32
ZnO	1.0 mg	395.82 ± 78.47**	11.44 ± 8.65**	0.98 ± 1.28	1.73 ± 1.66	4.20 ± 3.36
Control (Triton 0.1%)		5.36 ± 5.77	0.62 ± 0.38	0.86 ± 0.78	0.38 ± 0.85	0.67 ± 0.75
SWCNT	0.2 mg	47.33 ± 12.75**	61.63 ± 16.66**	29.58 ± 43.26**	11.98 ± 9.86**	8.74 ± 3.62**
SWCNT	0.4 mg	39.96 ± 19.05**	94.93 ± 37.00**	270.52 ± 132.86*	19.69 ± 21.59**	16.19 ± 14.49*
Control (Triton 0.05%)		0 ± 0	0.07 ± 0.15	0.11 ± 0.24	0.18 ± 0.41	0.41 ± 0.93
MWCNT (Short)	0.2 mg	9.22 ± 10.34*	0.14 ± 0.30	0.51 ± 0.86	0.44 ± 0.46	0.14 ± 0.32
MWCNT (Short)	1.0 mg	37.26 ± 36.95**	4.61 ± 6.57	26.23 ± 33.10**	2.25 ± 2.70	0.69 ± 1.55
Control (Triton 0.05%)		0.55 ± 0.60	0.14 ± 0.31	0.12 ± 0.27	0.61 ± 0.86	1.17 ± 1.49
MWCNT (Long)	0.2 mg	25.80 ± 12.18**	3.49 ± 1.94**	3.53 ± 0.85**	4.00 ± 3.64*	0.97 ± 1.07
MWCNT (Long)	0.6 mg	45.93 ± 13.04**	6.29 ± 3.46**	6.35 ± 7.53*	2.42 ± 1.58	0.91 ± 0.64
Control (Tween80 0.1%)		0.13 ± 0.30	0.17 ± 0.38	0.43 ± 0.48	0.90 ± 1.08	0.20 ± 0.45
Toner	1.0 mg	29.02 ± 11.53**	4.17 ± 4.75	2.27 ± 0.81*	1.38 ± 0.86	0 ± 0
(B) Inhalation exposure		3 days		1 month	3 months	
Pathological featurefeature						
control		–		–	–	
NiO	Low	–		–	–	
NiO	High	- or +		+	–	
control		–		–	–	
TiO_2_ (Rutile)	Low	–		–	–	
TiO_2_ (Rutile)	High	–		–	–	
control		–		–	–	
CeO_2_	Low	–		–	–	
CeO_2_	High	–		+	–	
control		–		–	–	
ZnO	Low	–		–	–	
ZnO	High	-~±		–	–	
Neutrophil counts in BALF (×1000 cells/mL ± SD)						
control		1.09 ± 2.43		14.23 ± 22.90	0.35 ± 0.77	
NiO	Low	1.39 ± 3.12		3.67 ± 2.84	1.30 ± 1.44	
NiO	High	84.10 ± 54.54**		21.97 ± 12.84	1.29 ± 1.43	
control		0 ± 0		0.09 ± 0.19	0 ± 0	
TiO_2_ (Rutile)	Low	0.15 ± 0.20		0 ± 0	0.48 ± 1.07	
TiO_2_ (Rutile)	High	0.12 ± 0.27		0.18 ± 0.19	0 ± 0	
control		0.55 ± 0.54		1.65 ± 0.64	1.94 ± 1.10	
CeO_2_	Low	38.22 ± 6.21**		19.70 ± 7.38**	11.13 ± 2.77**	
CeO_2_	High	96.74 ± 40.54**		114.92 ± 72.26**	49.18 ± 16.35**	
control		2.24 ± 1.95		0.56 ± 0.62	1.77 ± 0.66	
ZnO	Low	1.91 ± 0.63		0 ± 0*	2.50 ± 0.49	
ZnO	High	126.06 ± 45.21**		0.99 ± 2.17	2.24 ± 0.81	

*Grade of changes* - none, ± minimum, + mild,++ moderate, +++ remarked, *SD* standard deviation, Asterisks indicate significant differences compared with each control (Mann-Whitney U test) (*p < 0.05, **p < 0.01)

### MPO protein concentration in BALF

**Fig. 1:**
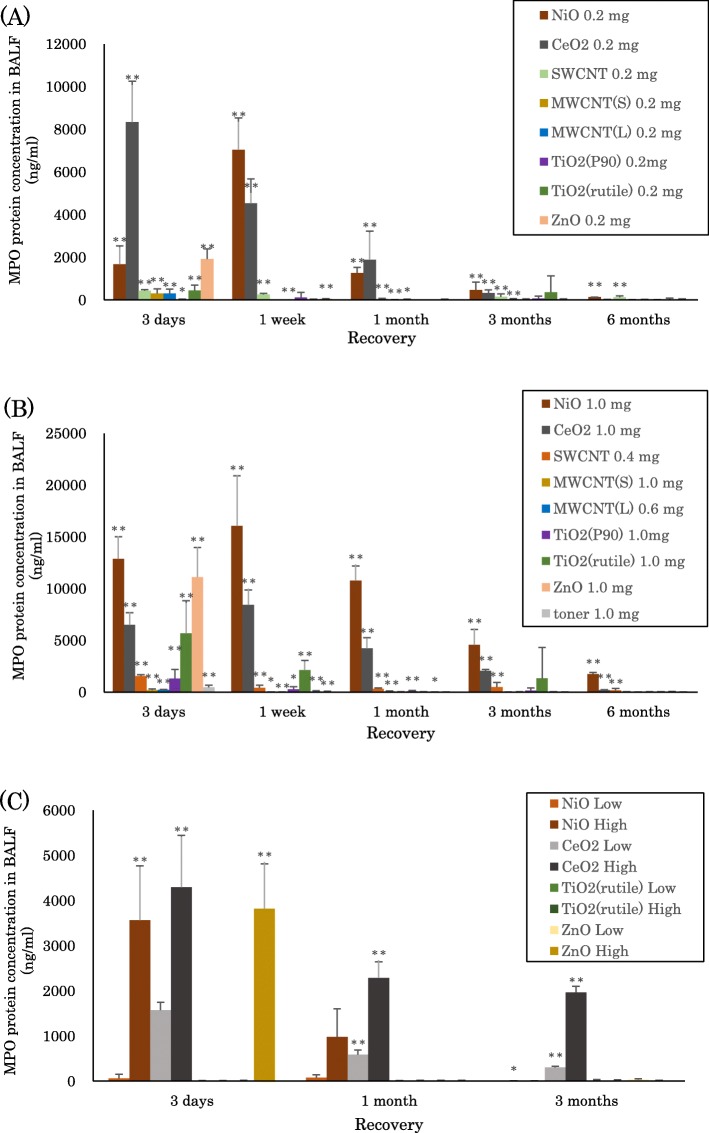
Concentration of MPO in BALF exposed to inhaled chemicals. **a** 0.2 mg exposed chemicals following intratracheal instillation. **b** 0.4–1.0 mg exposed chemicals following intratracheal instillation. **c** all of the exposed groups following inhalation. Error bar means standard deviation. Asterisks indicate significant differences compared with each control (Mann-Whitney U test) (*p < 0.05, **p < 0.01)

Figure 1a and b show the concentrations of MPO proteins in the BALF at each time point after intratracheal instillation of NiO, CeO_2_, SWCNT, MWCNT (S), MWCNT (L), TiO_2_ (P90), TiO_2_ (Rutile), ZnO, and toner. In the groups with low dose (0.2 mg) exposure to NiO, CeO2, MWCNT (S), MWCNT (L), and SWCNT, increases in the concentration of MPO protein were observed until 3 months to 6 months. In the high dose (0.4–1.0 mg) exposures, the concentration of MPO increased persistently and in a dose-dependent manner compared to the low dose exposures. In the exposure to TiO_2_ (P90), TiO_2_ (Rutile), ZnO, and toner, the concentration of MPO protein increased mainly at 3 days and 1 week. Even in the high dose (0.4–1.0 mg) exposure, there was a tendency of transient increase in the concentration of MPO.

Figure 1c shows the concentrations of MPO proteins in BALF at each time point after the inhalation of NiO, CeO2, TiO_2_ (Rutile), and ZnO. In the NiO- high concentration group, the MPO increased at 3 days and 1 week. In the CeO_2_- low and high concentration groups, increases in the concentration of MPO were observed until 3 months. There was no significant increase in MPO concentration, however, in the group of ZnO and TiO_2_ (Rutile) exposure, except for the ZnO- high concentration group at 3 days after inhalation.

### Correlation between MPO and inflammatory markers

**Fig. 2:**
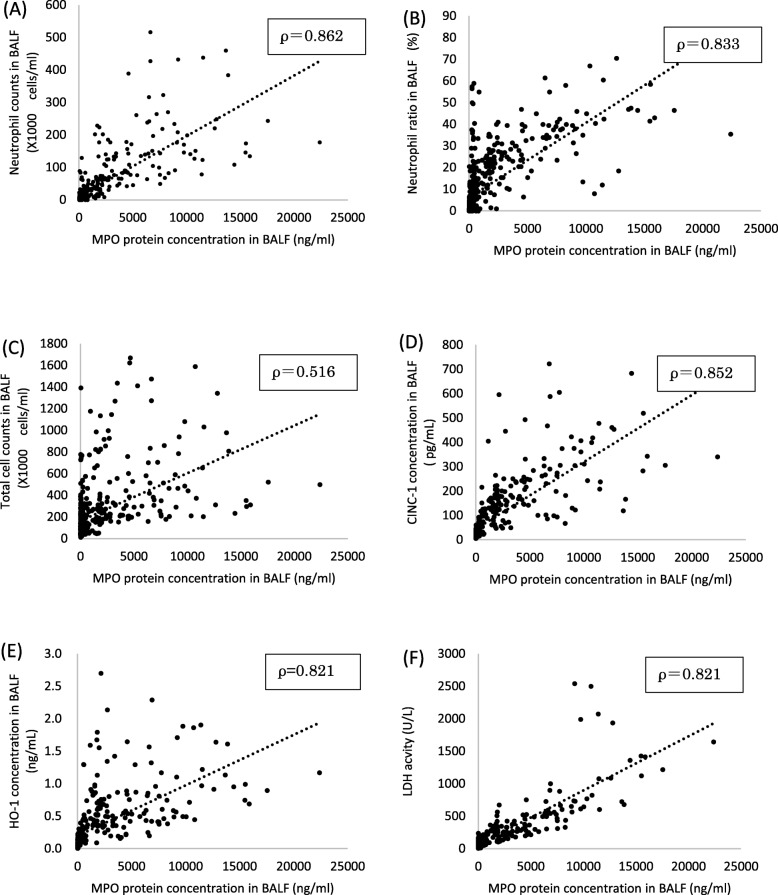
Relationship between MPO and inflammatory markers: **a** neutrophils, **b** percent of neutrophils in total cells, **c** total cell, **d** CINC-1, **e** HO-1 and **f** LDH versus MPO concentration in BALF after inhalation and intratracheal instillation of inhaled chemicals. Values of ρ are Spearman’s rank correlation coefficient for all the data

Figure 2 and Additional file 1 (Figure S1-S2) shows the correlation between the concentration of MPO proteins and inflammatory markers. The concentration of MPO protein in BALF correlated well with the total cell counts, neutrophil count, neutrophil ratio, the concentration of rat cytokine-induced neutrophil chemoattractant (CINC)-1, the concentration of rat heme oxygenase (HO)-1, and the activity of released lactate dehydrogenase (LDH).

### MPO immunostaining

**Fig. 3:**
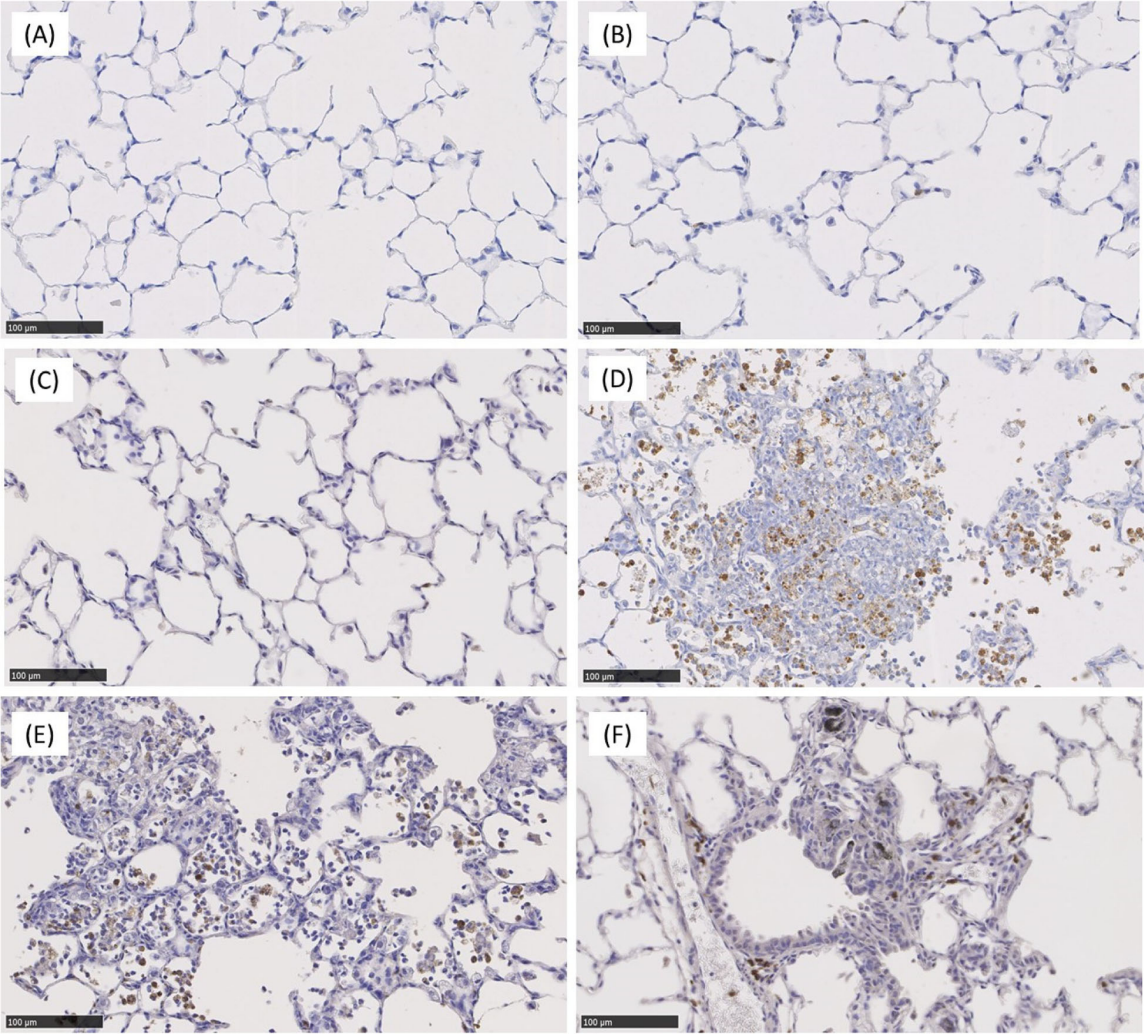
Myeloperoxidase immunostaining of lung sections at 1 month exposure: (**a**) distilled water as a negative control, (**b**) 1 mg TiO_2_ (Rutile)-exposed lung, (**c**) 1 mg ZnO-exposed lung, (**d**) 1 mg NiO-exposed lung, (**e**) 1 mg CeO_2_-exposed lung, (**f**) 0.6 mg MWCNT (L)-exposed lung. Positive areas were observed mainly at the gathering sites of inflammatory cells at 1 month in NiO-, CeO_2_- and MWCNT (L)- exposed groups, and no positive areas were observed in negative control, TiO_2_ (Rutile) and ZnO- exposed groups

Figure 3 shows MPO immunostaining in the high dose of TiO_2_ (Rutile)-, ZnO-, NiO-, CeO_2_- and MWCNT (L)- exposure groups and a negative control group (distilled water) at 1 month after intratracheal instillation. MPO was observed mainly at the gathering sites of inflammatory cells at 1 month in the NiO-, CeO_2_- and MWCNT (L)- high exposure group, and there were MPO positive in some of the macrophages that phagocytosed neutrophils in the NiO-, CeO_2_- and MWCNT (L)- groups as well.

### Assessment of the accuracy of MPO measurement of the toxicity of chemicals

**Fig. 4:**
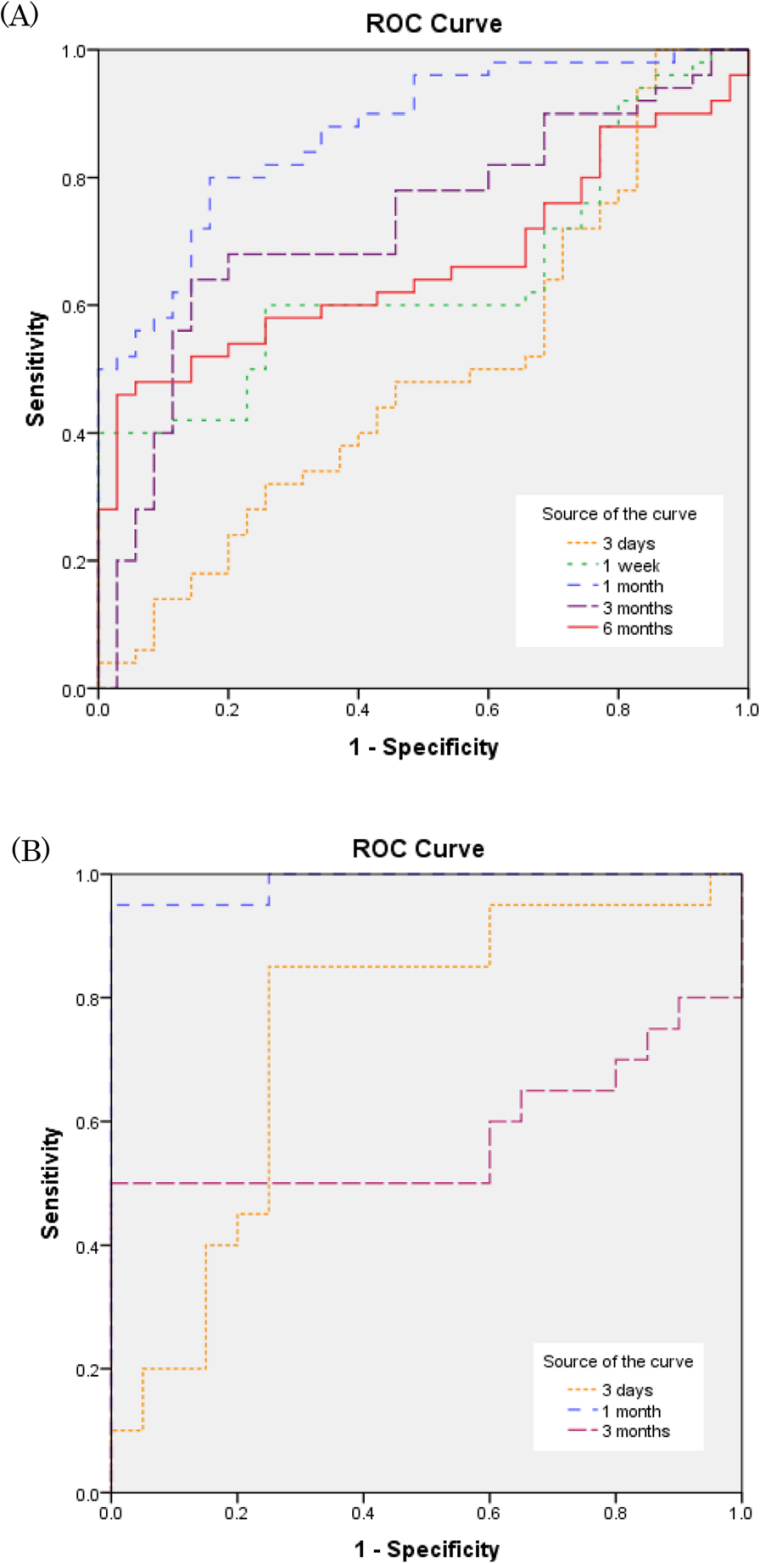
The receiver operating characteristics for the toxicity of chemicals by the concentration of MPO proteins. **a** Intratracheal instillation. **b** Inhalation exposure. The area under the curves at 1 months after exposure following both of intratracheal instillation and inhalation were larger than the other observation times

Figure 4 shows the results of the receiver operating characteristics (ROC) for the toxicity of chemicals by the concentration of MPO proteins in the intratracheal instillation and inhalation exposure. The largest areas under the curves (AUC) using ROC curves for the toxicity of chemicals in intratracheal instillation and inhalation exposure were 0.87 (95% CI, 0.80–0.94) and 0.99 (95% CI, 0.96–1), respectively. The largest AUCs in both examinations were observed at 1 month after exposure (Table 3).

**Table 3:** The values of AUCs for the toxicity of chemicals by the concentration of MPO proteins

(A) Intratracheal instillation				(B) Inhalaton exposure			
Time	AUC	95%CI	*p* value	Time	AUC	95%CI	*p* value
3 days	0.495	0.37–0.62	0.943	3 days	0.748	0.59–0.91	0.007
1 week	0.647	0.53–0.76	0.022	1 month	0.988	0.96–1.00	0.000
1 month	0.871	0.80–0.94	0.000	3 months	0.580	0.38–0.78	0.387
3 months	0.727	0.62–0.84	0.000				
6 months	0.665	0.55–0.78	0.010				

## Discussion

In this study, NiO, CeO_2_, MWCNT (S), MWCNT (L), and SWCNT were classified as chemicals with high pulmonary toxicity, and TiO_2_ (P90), TiO_2_ (Rutile), ZnO and toner were classified as chemicals with low toxicity. It has been reported that inhaled toxic chemicals such as silica and asbestos cause persistent inflammation, irreversible fibrosis and tumor [9, 11, 12, 25, 26]. Some reports have shown that exposure to NiO, SWCNT, and MWCNT, considered to have high levels of pulmonary toxicity, also induce persistent inflammation, irreversible fibrosis and tumor [27–32]. It has also been reported that long term inhalation exposure to NiO or MWCNT induces lung tumor in rats and that they have pulmonary carcinogenicity [29, 33]. In exposure to low toxicity TiO_2_, ZnO and toner, on the other hand, transient or no inflammation and no irreversible changes were observed [12, 34–36]. Moreover, in studies of inhalation exposure for 2 years the toner and TiO_2_ did not significantly induce lung tumor in a rat model [12, 36]. From this, we can conclude that our classification of toxicity corresponds to the data of other researchers.

In the present study, 1 mg/rat was used as the maximum dose for the intratracheal instillation. We previously reported that doses in excess of 1 mg/rat induced pulmonary inflammation and delay of the biological half time of nanoparticles [37]. It has been reported in toner studies as well that the clearance delay of alveolar macrophages occurs between 1 and 3 mg of lung deposition in rat [38, 39], indicating that the threshold of overload is between 1 and 3 mg. From these data, we speculated that exposure to doses in excess of 1 mg/rat might induce not only pulmonary toxicity by the chemicals themselves but also toxicity from the excessive dose. We used the following formula to estimate what amount in human exposure corresponds to the intratracheal instillation dose in rat.

(Deposited mass of particles) = (exposure concentration of particles).

× (tidal volume) × (breathing frequency).

× (exposure hours in day) × (days of exposure).

× (particle deposition fraction) (1).

Assuming that inhaled chemicals would be deposited at the same rate (particle deposition efficiency 0.1, amount of deposited material /1 g of lung weight) in rats and humans, we estimated that the exposure time per human would be 463 days (calculation in rat and human under assumption of tidal volume 2.1 and 625 mL/ times; breathing frequency volume 102 and 12 times/min; exposure hours in day 8 h), if 1 mg/rat as the lung burden was converted into human exposure at a concentration of 3 mg/m^3^, which the American Conference of Governmental Industrial Hygienists (ACGIH) defined as the threshold limit values-time weighted average (TLV-TWA) of respirable dust. We think that 1 mg/rat as the lung burden of inhaled material by intratracheal instillation may correspond to approximately 1.8 years of inhalation exposure for humans at a concentration of 3 mg/m^3^ (working time 8 h/day, 5 days/week).

In the inhalation exposure in this study, on the other hand, the common exposure concentration was about 2 mg/m^3^ and the maximum exposure concentration was about 10 mg/m^3^. We calculated that, in inhalation exposure for 1 month, the lung burdens of rat at 2 mg/m^3^ and 10 mg/m^3^ were 0.31 mg/lung and 1.54 mg/lung, respectively. We considered that the lung burden in inhalation exposure to nanoparticles might not be very different from the same doses in intratracheal instillation under the same experimental conditions as in the present inhalation study.

Whether MPO can be a useful biomarker of pulmonary toxicity depends on whether it can reflect the persistent lung inflammation. Intratracheal instillation of NiO, CeO_2_, MWCNT (S), MWCNT (L), and SWCNT induced persistent increases in MPO protein concentration (Fig. 1a, b) and caused persistent inflammation or fibrosis in BALF or other pathological features, suggesting that MPO reflects pulmonary toxicity. Inhalation exposure to NiO and CeO_2_ elevated the concentration of MPO from 1 month to 3 months, but inhalation of ZnO at a high concentration increased MPO at 3 days only (Fig. 1c). In the present study, transient inflammation in the ZnO exposure was observed not only in the intratracheal instillation but also in the inhalation exposure. We suggest that the reason for the transient increase in the concentration of MPO in the ZnO exposure was that ZnO has high solubility and that the release of Zn ion in large quantities in the lung induces transient inflammation [21]. Adamcakova-Dodd et al [35] reported that 100% of ZnO nanoparticles dissolved within the first 24 h of mixing in an artificial interstitial fluid (pH 4.5), and recognized that ZnO has high solubility. Similarly, copper oxide nanoparticles, which are considered to have high solubility, were reported to induce inflammation at an acute phase in the lung through dissolution [40]. Therefore we considered that ZnO, with its high solubility, induced hardly any inflammation at the chronic phase and may have low toxicity.

We examined the sensitivity and specificity of MPO as a biomarker for the evaluation of pulmonary toxicity by conducting ROC analysis in the intratracheal instillation and the inhalation exposure (Fig. 4). The highest values of AUCs in the intratracheal instillation and in the inhalation exposure were 0.871 (95% CI, 0.80–0.94) and 0.988 (95% CI, 0.96–1.00) respectively, and both appeared at 1 month after exposure. The results of the ROC analysis were interpreted as follows: AUC < 0.70, low diagnostic accuracy; AUC in the range of 0.70–0.90, moderate diagnostic accuracy; and AUC ≧0.90, high diagnostic accuracy [41]. Therefore we surmise that the concentration of MPO at 1 month could most accurately reflect the rank of pulmonary toxicity. The reason why it is difficult to evaluate the pulmonary toxicity of inhaled chemicals at an acute phase is that a bolus effect occurs in intratracheal instillation, in which there is not only the toxicity of the material itself but also, at the same time, the negative effect of exposure. The difference in pulmonary toxicity or inflammation may be difficult to evaluate in the acute phase. In the inhalation study, also, pulmonary toxicity could be better evaluated at 1 month after exposure than at earlier points.

To investigate the relationship between MPO and lung disorder due to pulmonary inflammation, we examined in BALF samples the correlation between MPO and total cell number, neutrophil count, CINC-1, which is an inflammatory chemokine, HO-1, which is an oxidative stress marker, and LDH, which is a cytotoxic factor. There were good correlations between MPO and total cell counts, neutrophil counts, CINC-1, HO-1, and LDH in the BALF (Fig. 2). There are some reports in which inflammation related markers increased lung inflammation by inhaled chemicals [26, 42, 43]. Stringer et al [42] reported that cigarette particles caused the upregulation of CINC-1 expression and activation of MPO in a rat exposure model. Knaapen et al [43] reported an increase in total cell counts, neutrophils, LDH, and MPO concentration in an intratracheal instillation rat model using crystalline silica (DQ12). Since the relationships between MPO and these markers were observed in our study as well, it is thought that there is a series of steps following the migration of neutrophils, activation of neutrophils, MPO release by neutrophils, and lung injury. On the other hand, Haegens et al [44] reported that neutrophil influx in lipopolysaccharide (LPS)-exposed MPO deficient mice were significantly decreased compared with wild-type (WT) mice, and LPS-exposed MPO deficient mice demonstrated a decrease pattern of inflammatory cytokine (interleukin (IL)-6) and chemokine (Macrophage Inframmatory Protein (MIP)-1α, regulated on activation, normal T cell expressed and secreted (RANTES)) expression. They suggest that MPO not only plays an important role in the infiltration of lung neutrophilia but also indirectly contributes to chemokine and cytokine production that may govern inflammatory processes. It has been reported that carbon nanotube or asbestos exposed MPO deficient mice has a milder lung inflammation in the acute phase than WT mice as well [14, 45], suggesting that MPO were involved in the process of pulmonary inflammation by nanomaterials and accelerated the inflammation. Actually, observing lung pathological specimens by MPO immunostaining revealed that MPO was positive mainly at the gathering site of inflammatory cells. The positive areas of MPO staining were mainly neutrophils and macrophages that phagocytosed neutrophils at inflammatory lesions such as the granulomatous area at 1 month in the NiO-, CeO_2_- and MWCNT (L)- high exposure groups, also suggesting that MPO is involved in lung inflammation (Fig. 3).

## Conclusion

In this study, we focused on MPO, which has the ability to directly damage cells via oxidative stress, and examined its usefulness as a biomarker for evaluating the pulmonary toxicity of nanoparticles, using inhaled chemicals including nanomaterials which were classified as having pulmonary toxicity. The results of the measurement of MPO protein concentration reflected that the most accurate ranking of pulmonary toxicity in both intratracheal instillation and inhalation exposure was at 1 month after exposure. MPO correlated with inflammatory cells, other inflammatory chemokines, and an oxidative stress marker, and it was thought that MPO was related to lung disorder due to pulmonary inflammation by nanomaterials. Taken together, we suggest that MPO can be a useful biomarker for the ranking of pulmonary toxicity of nanomaterials in both intratracheal instillation and inhalation exposure.

## Methods

### Sample nanomaterials

Commercially provided NiO (US3355, US Research Nanomaterials, Houston, TX), TiO_2_ (P90) (Aeroxide Evonik Degussa Corp, Nordrhein-Westfalen, Germany), TiO_2_ (Rutile) (MT-150AW, Teyca Co. Ltd., Osaka, Japan), CeO_2_ (Wako Chemical, Ltd. Japan), and ZnO (Sigma-Aldrich Co. LLC., Tokyo, Japan) were dispersed in 0.4 ml distilled water [17–21].

SWCNT and MWCNTs synthesized by the catalytic chemical vapor deposition (CVD) method were provided by Nikkiso Co., Ltd., Tokyo. The MWCNT (S) consisted of cut up short fibers from MWCNT (L). The preparation of MWCNT (S) was shown in a previous study [22]. Triton X-100 purchased from Wako Pure Chemical Industries, Ltd. (Japan) was used as a dispersant for the preparation of the suspensions. SWCNT, MWCNT (S) and MWCNT (L) were suspended in 0.4 ml distilled water including 0.1%, 0.05 0.05% Triton X-100, respectively [22–24].

The test toner was provided by Fuji Xerox Co., Ltd., Tokyo, Japan, as an experimental toner sample used exclusively for the planned intratracheal instillation study. The toner was synthesized by dispersed toner components in the liquid phase and covered mechanically and electrostatically with TiO2 nanoparticles and amorphous silica nanoparticles as the external additive. The test toner was suspended with 0.4 ml distilled water including Tween 0.1% [25].

### Animals

Male Fischer 344 rats (9–11 weeks old) used in the exposure to NiO, CeO_2_, TiO_2_ (Rutile), and ZnO were purchased from Charles River Laboratories International, Inc. (Japan). Male Wistar Hannover rats (11 weeks old) used in the exposure to TiO_2_ (P90) were purchased from Japan SLC, Inc. (Shizuoka, Japan). Male Wistar rats (8 and 9 weeks old) used in the exposure to SWCNT and MWCNTs were purchased from Kyudo Co., Ltd. (Kumamoto, Japan). Female Wistar rats (8 weeks old) used in the exposure to toner were purchased from Kyudo Co., Ltd. (Kumamoto, Japan). The animals were kept in the Laboratory Animal Research Center of the University of Occupational and Environmental Health for 2 weeks with access to free-feeding of commercial diet and water. All procedures and animal handling were done according to the guidelines described in the Japanese Guide for the Care and Use of Laboratory Animals as approved by the Animal Care and Use Committee, University of Occupational and Environmental Health, Japan.

### Intratracheal instillation

The NiO, TiO_2_ (P90), TiO_2_ (Rutile), CeO_2_, and ZnO nanoparticles were suspended in 0.4 ml distilled water. A 0.2 mg (low dose) or 1 mg (high dose) was administered to rats (12 weeks old) in a single intratracheal instillation. The MWCNTs were suspended in 0.4 ml distilled water including 0.05% Triton X-100. Rats were instilled once at a dose of 0.2 mg (low dose) or 1 mg (high dose) of MWCNT (S), and at a dose of 0.2 mg (low dose) or 0.6 mg (high dose) of MWCNT (L). The 0.2 mg (low dose) or 0.4 mg (high dose) of SWCNT was suspended in 0.4 ml of distilled water including 0.1% Triton X-100. The test toner was suspended with 0.4 ml distilled water including 0.1% Tween-80. 1 mg (3.3 mg/kg) of toner was instilled once to rats. Each of the negative control groups received dispersion mediums which used the suspensions in each exposure examination. Animals were dissected at 3 days, 1 week, 1 month, 3 months and 6 months after the instillation.

### Inhalation exposure

The setup used here has been described in more detail in our previous papers [46, 47]. Briefly, an aerosol generation system consisting of a pressurized nebulizer (Nanomaster, JSR Corp., Tokyo, Japan) and a drying section was connected to a whole body exposure chamber with rat cages. The inhalation exposure was conducted by supplying aerosol particles of NiO, CeO_2_, TiO_2_ (Rutile) and ZnO at two concentrations. Nanoparticles of the suspensions were diluted with water and set in the nebulizer to be sprayed with compressed air at a flow rate of 40 L/min. As a drying process, the droplets were successively passed through a heated (150 °C) tube to remove the water. After the drying process, air containing bipolar ions supplied by an ionizer (SJ-M, Keyence Corp., Tokyo, Japan) was introduced at a flow rate of 10 L/min, concurrently with the aerosol flow, to neutralize the aerosol particles and to reduce particle wall loss in the tubing caused by electrostatic forces. Clean air was added to the resulting aerosol flow to set the total airflow rate to 100 L/min, and the aerosol was fed through the exposure chamber for 6 h on each day of the 4 week inhalation test. The size and number concentration of the aerosol particles inside and outside the exposure chamber were analyzed using a particle size spectrometer (model 1000XP WPS, MSP Corp., Shoreview, MN, USA) built for in-line monitoring. For comparison, the aerosol particles were sampled by an electrostatic precipitator for off-line analysis using field emission scanning electron microscopy (FE–SEM; S-5200, Hitachi High Technologies Corp., Tokyo, Japan). In addition, the mass concentration of the aerosol in the chamber was measured by a gravimetric method wherein the aerosol was admitted through fibrous filters and the collected particles were weighed.

Each of the aerosol samples exhibited very stable particle size distributions over 6 h on each day of the entire period of the inhalation test. The particle size distributions showed two peaks at around 30 and 100 nm for the NiO aerosols and around 30 and 200 nm for the TiO_2_ aerosols, which was due to Coulomb explosion [47]. The particle size distributions of the CeO_2_ and ZnO aerosols displayed peaks at around 90 nm and 120 nm, respectively. The average mass concentrations of the aerosols measured daily for the 4 weeks were 0.32 ± 0.07 or 1.65 ± 0.20 mg/m^3^ for NiO, 2.09 ± 0.34 or 10.2 ± 1.38 mg/m^3^ for CeO_2_, 0.50 ± 0.26 or 1.84 ± 0.74 mg/m^3^ for TiO_2_, and 2.11 ± 0.54 or 10.4 ± 1.39 mg/m^3^ for ZnO.

### Animals following inhalation and intratracheal instillation

In the exposure to NiO, TiO_2_ (P90), TiO_2_ (Rutile), CeO_2_, ZnO, and the control, there were 10 rats in each group, divided into two subgroups of five animals in each low dose and high dose group at each time course. In each of the first subgroups, 5 rats provided bronchoalveolar lavage in each time course. The lungs were inflated with 20 mL physiological saline under a pressure of 20 cm water, and BALF was collected from whole lung divided two to three times. Between 15 and 18 mL of BALF was collected in collection tubes by free fall. In the second subgroups, the lung was divided into right and left lungs, and histopathological evaluation was performed with the left lung inflated and fixed by 10% formaldehyde.

In the exposure to SWCNT, MWCNTs, and toner, there were 10 rats each in the control, low dose and high dose groups at each time course. The BALF was collected using physiological saline that was poured through a cannula inserted into the respiratory tract in the right lung while the left lung was clamped. 3–10 ml (different volumes of lavage fluid were based on animal ages) of physiological saline was infused at each time point, and up to 50 ml of lavage fluid was collected in total. The left lung was inflated and fixed by 10% formaldehyde in the MWCNTs studies and 4% paraformaldehyde in the studies of SWCNT and toner.

### Analysis of inflammatory cells in BALF with cytospin

The obtained BALF was centrifuged at 400 g at 4 °C for 15 min, and the supernatant was transferred to a new tube and frozen for measuring the cytokines. The pellets were washed by suspension with polymorphonuclear leukocyte (PMN) Buffer (137.9 mM NaCl, 2.7 mM KCl, 8.2 mM Na2HPO4, 1.5 mM KH2PO4, 5.6 mM C6H12O6) and centrifuged at 400 g at 4 °C for 15 min. After the supernatant was removed, the pellets were re-suspended with 1 mL of PMN Buffer. The number of cells in the BALF was counted by Celltac (Nihon Kohden Corp., Tokyo, Japan), and the cells were splashed on a slide glass using cytospin. After the cells were fixed and stained with Diff-Quik (Sysmex Corp., Hyogo, Japan), the number of neutrophils and alveolar macrophages were counted by microscopic observation.

### Measurement of myeloperoxidase, chemokines, lactate dehydrogenase, and heme oxigenase-1 in BALF

The concentrations of rat MPO proteins in the BALF samples in all of the examinations were measured by ELISA kits, HK105 (Hycult Biotech, The Netherlands). The concentrations of CINC-1 in the BALF were measured by ELISA kits, #RCN100 (R& D Systems, Minneapolis, MN), and the concentrations of HO-1 in the NiO, TiO_2_ (P90), TiO_2_ (Rutile), CeO_2_, and ZnO exposure examinations were measured by an ELISA kit, ADI-EKS-810A (Enzo Life Sciences, Farmingdale, NY). The activity of LDH in the NiO, TiO_2_ (Rutile), CeO_2_, and ZnO exposure examinations was measured by a Cytotoxicity Detection Kit^PLUS^ (LDH) (Roche Diagnostics GmbH, Mannheim, Germany). All measurements were performed according to the manufacturer’s instructions.

### Histopathology

The obtained lung tissue, which was inflated and fixed with 10% formaldehyde or 4% paraformaldehyde under a pressure of 25 cm water, was embedded in paraffin, and 5-mm-thick sections were cut from the lobe, then stained with hematoxylin and eosin. Activation of neutrophil inflammation was evaluated by myeloperoxidase immunostaining using the lung tissue samples of 1 month after intratracheal instillation of TiO_2_ (Rutile), ZnO, NiO, CeO_2_ and MWCNT (L).

### Statistical analysis

Statistical analysis was carried out using the Mann-Whitney test with differences of *p* < 0.05 considered to be statistically significant. (SPSS, SPSS Inc., Chicago, IL, USA). Construct validity was measured using Spearman’s rank correlation coefficients between the concentration of MPO protein, neutrophil counts, neutrophil ratio, total cell counts, the concentration of CINC-1, concentration of HO-1, and activity of released LDH. We assigned the toxicity of exposed nanomaterials of which high or low to the values of MPO protein concentration of each sample, and analyzed with SPSS software the sensitivity and specificity for high toxicity at each time points to create the ROC curves and AUCs.

## Additional file

Additional file 1: **Figure S1-S2.** Relationship between MPO and inflammatory markers after intratracheal instillation or inhalation exposure. Figure S1: Relationship between MPO and inflammatory markers: (**a**) neutrophils, (**b**) percent of neutrophils in total cells, (**c**) total cell, (**d**) CINC-1, (**e**) HO-1 and (**f**) LDH versus MPO concentration in BALF after intratracheal instillation of inhaled chemicals. Values of ρ are Spearman’s rank correlation coefficient for each of the data. **Figure S2.** Relationship between MPO and inflammatory markers: (**a**) neutrophils, (**b**) percent of neutrophils in total cells, )**c**) total cell, (**d**) CINC-1, (**e**) HO-1 and (**f**) LDH versus MPO concentration in BALF after inhalation exposure. Values of ρ are Spearman’s rank correlation coefficient for each of the data. (DOCX 141 kb)
