# Supplementary material for: Usefulness of myeloperoxidase as a biomarker for the ranking of pulmonary toxicity of nanomaterials
Source: Part Fibre Toxicol. 2018 Oct 23;15:41. doi: 10.1186/s12989-018-0277-x (PMC6199695; doi:10.1186/s12989-018-0277-x)
Supplement: Supplementary file 1 — Figure S1-S2. Relationship between MPO and inflammatory markers after intratracheal instillation or inhalation exposure. Figure S1: Relationship between MPO and inflammatory markers: (a) neutrophils, (b) percent of neutrophils in total cells, (c) total cell, (d) CINC-1, (e) HO-1 and (f) LDH versus MPO concentration in BALF after intratracheal instillation of inhaled chemicals. Values of ρ are Spearman’s rank correlation coefficient for each of the data. Figure S2. Relationship between MPO and inflammatory markers: (a) neutrophils, (b) percent of neutrophils in total cells, )c) total cell, (d) CINC-1, (e) HO-1 and (f) LDH versus MPO concentration in BALF after inhalation exposure. Values of ρ are Spearman’s rank correlation coefficient for each of the data. (DOCX 141 kb) [file 12989_2018_277_MOESM1_ESM.docx]

Figure S1

Figure S2
